# Three dimensional and four dimensional live imaging to study mechanisms of progressive neurodegeneration

**DOI:** 10.1016/j.jbc.2024.107433

**Published:** 2024-05-31

**Authors:** Jeremy W. Linsley, Terry Reisine, Steven Finkbeiner

**Affiliations:** 1 Center for Systems and Therapeutics, Gladstone Institutes, San Francisco, California, USA; 2 Operant Biopharma, San Francisco, California, USA; 3 Independent Scientific Consultant, Santa Cruz, California, USA; 4 Taube/Koret Center for Neurodegenerative Disease, Gladstone Institutes, San Francisco, California, USA; 5 Departments of Neurology and Physiology, University of California, San Francisco, California, USA; 6 Neuroscience Graduate Program, University of California, San Francisco, California, USA; 7 Biomedical Sciences Graduate Program, University of California, San Francisco, California, USA

**Keywords:** neurodegenerative disease, organoids, two photon, light sheet, neurodegenerative disease models, biosensors, fluorescence microscopy, Alzheimer's disease, Parkinson's disease, ayotrophic Lateral Sclerosis, robotic microscopy

## Abstract

Neurodegenerative diseases are complex and progressive, posing challenges to their study and understanding. Recent advances in microscopy imaging technologies have enabled the exploration of neurons in three spatial dimensions (3D) over time (4D). When applied to 3D cultures, tissues, or animals, these technologies can provide valuable insights into the dynamic and spatial nature of neurodegenerative diseases. This review focuses on the use of imaging techniques and neurodegenerative disease models to study neurodegeneration in 4D. Imaging techniques such as confocal microscopy, two-photon microscopy, miniscope imaging, light sheet microscopy, and robotic microscopy offer powerful tools to visualize and analyze neuronal changes over time in 3D tissue. Application of these technologies to *in vitro* models of neurodegeneration such as mouse organotypic culture systems and human organoid models provide versatile platforms to study neurodegeneration in a physiologically relevant context. Additionally, use of 4D imaging *in vivo*, including in mouse and zebrafish models of neurodegenerative diseases, allows for the investigation of early dysfunction and behavioral changes associated with neurodegeneration. We propose that these studies have the power to overcome the limitations of two-dimensional monolayer neuronal cultures and pave the way for improved understanding of the dynamics of neurodegenerative diseases and the development of effective therapeutic strategies.

Most neurodegenerative diseases are progressive, manifesting over decades, resulting in gradual dysfunction and ultimately death of select neuronal populations in the central nervous system (CNS). This slow time course makes modeling human neurodegenerative diseases difficult, as does the complexity of the human nervous system. Traditional imaging studies have been done in cells, tissues, or organisms at single time points attempting to identify and define molecular hallmarks and etiologies of neurodegenerative diseases. However, these static images often fail to capture the context of the slow time course and the heterogeneity of structural and functional changes leading to degeneration. Recent advances in positron emission tomography (reviewed in (1)) and functional magnetic resonance (2, 3, 4, 5) have facilitated monitoring of the development and progression of neurodegenerative disease across prolonged timescales with much success, though without cellular resolution. Other recent technological advances in cellular microscopy imaging methods are now enabling scientists to monitor neurons in 3-dimensions (3D) and over prolonged time courses, providing 4-dimensional (4D) analysis to better capture the time-dependence and spatial context of neurodegenerative disease at a cellular level. This review will focus on advances in microscopic 4D analysis of neurodegeneration and technological developments that enable the elucidation of neurodegenerative mechanisms at a cellular and molecular level. These approaches have the potential to provide a foundation for the development of novel therapeutics that may be more effective than those that have been developed using static analyses, which have generally failed in clinical studies.

Much of what we know about neurodegeneration at a cellular level has been learned from microscopy imaging studies of two-dimensional (2D) monolayer neuronal cultures, either derived from animals or human induced pluripotent stem cells (iPSCs). These approaches have clear limitations. Firstly, cells in dissociated cultures lack the natural organization of neurons in brain essential to understand how neuronal circuitry slowly changes over time. Secondly, 2D neuron cultures lack an appropriate representation of non-neuronal cells, including astrocytes and microglia, which normally support the development, function, health, and immunity of neurons and may also be a driving force in the progression of many neurodegenerative diseases. Finally, these 2D cultures are incapable of modeling the transmission of misfolded proteins such as tau or alpha-synuclein (aSYN) that are responsible for the patterned spread of pathology throughout the brain in some neurodegenerative diseases.

Here, we will discuss the advances in microscopy technology that are now making it possible to ask and answer questions in 3D cellular systems and the multiple types of 3D models of neurodegeneration amenable to live cell imaging. These systems include transparent animal models such as zebrafish larvae, as well as rodents studied *in vivo* or *in vitro* in tissue slices as organotypic cultures (Table 1). The *in vivo* animal models of neurodegenerative diseases have the advantage of providing insight into the links between early stages of dysfunction and alterations in animal behaviors that may be analogous to signs of disease in humans. However, animal models are often based on genetic perturbations linked to rare forms of degenerative diseases, and therefore their relationship to more common sporadic forms of diseases are unclear. Furthermore, some features of neurodegeneration may be specific to humans and impossible to recapitulate in animal models. Therefore, patient iPSC-derived neurons (i-neurons) are powerful because they precisely replicate the genetic makeup of the patient from which they are derived in a way that is impossible to achieve in non-human models. This provides an opportunity to investigate the role of different endogenously expressed genes and genetic variants in neurodegeneration and the chance to model sporadic forms of disease. In addition, human i-neurons can be grown into 3D organoid models to recapitulate some of the circuitry and heterotypic interactions found *in vivo*. Since neurodegeneration and neuronal death are heterogeneous, 3D live imaging technologies provide the opportunity to understand how neurons resist degeneration at a single cell level even when their immediate neighbors begin to degenerate and die. This information can aid in identifying potential novel molecular targets to discover therapeutics to slow neurodegeneration and disease progression.

**Table 1:** 3D/4D biological models of neurodegeneration

Property	Mouse *in vivo*	Mouse organotypic	Zebrafish larvae	hiPSC organoids whole/sliced
Brain coverage	Low	Partial	Total	Low/total
Prep invasiveness	Medium	High	Low	Low/high
High throughput screening compatible?	No	Yes	Yes	Yes
Behavior correlate	Yes	No	Yes	No
Duration of imaging	Short	Long	Short	Long
Aged models available	Yes	Yes	No	No
Example references	(85)	(128)	(113)	(138)

### Imaging technologies to study neurodegeneration in 4D

There are enormous challenges in designing a technology capable of visualizing cellular and subcellular activities associated with degeneration within brain tissue. Brain tissue is optically opaque, and thus probing deep within it necessitates strong optical illumination and complex optics for dealing with light scattering. In addition, neurocircuitry and patterns of neurodegeneration often span large distances in the CNS, so visualization requires optical illumination that spans those long distances. However, strong optical illumination can generate phototoxicity and heat, which can lead to artifactual results or further contribute to neurodegeneration. For longitudinal imaging, the sample must remain stationary with respect to the optical objective across the region of interest, and the camera must maintain a frame rate of imaging that can catch relevant biological phenomena that occur over time scales at various orders of magnitude, from nanoseconds (action potentials (6)) to months (amyloid accumulation (7)). These limitations have been overcome to varying extents by the development and use of more advanced imaging technologies, including confocal, two-photon (2P) and light sheet microscopy, miniscope imaging, as well as robotic microscopy, which can be used for 4D image analysis of neurodegenerative disease models (Fig. 1).

**Figure 1:**
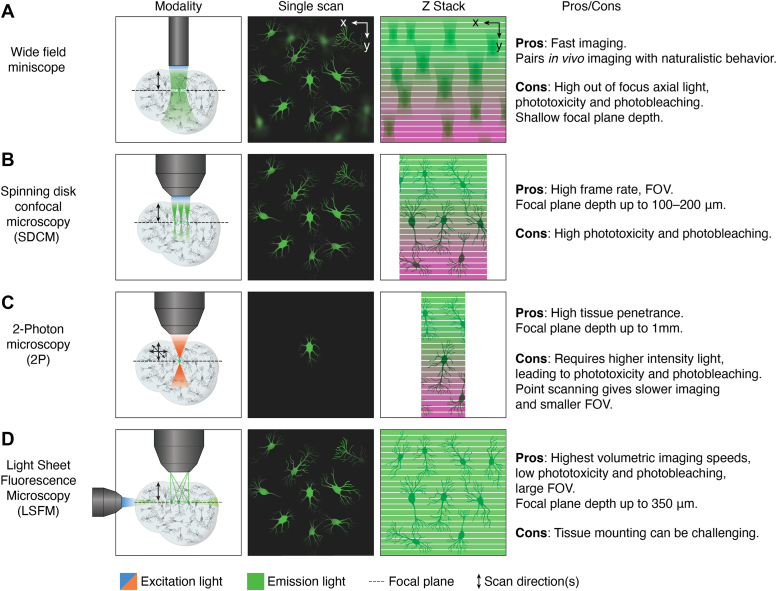
**Optical methodologies for 4D microscopy.** Different optical methodologies provide unique opportunities and insights. *A*, wide field miniscope imaging can be attached to the skull of an animal and used to generate fluorescent neuronal imaging *in vivo*. It suffers from out-of-focus axial light scatter, which distorts z-stack reconstruction, as well as the potential for phototoxicity and photobleaching (*magenta* bands) without careful control. *B*, spinning disk confocal microscopy (SDCM) uses pinholes to narrow light at the focal plane, facilitating optical sectioning down to around 1 to 200 μm of depth (8) and minimizing axial light scatter with high frame rate and large fields of view (FOV). It can also generate high phototoxicity and photobleaching, particularly at the end of a z-scan due to continued high energy illumination. *C*, 2-Photon microscopy (2P) uses 2 photons of infrared light to excite fluorescent probes deep within tissue (up to around 1 mm deep in brain tissue) (16). The use of infrared light facilitates deep tissue penetration, but the technique suffers from phototoxicity and photobleaching as well as a comparatively smaller field of view and slower imaging speeds. *D*, light sheet fluorescence microscopy (LSFM) uses perpendicular illumination to create a light sheet within the optical plane. This facilitates high speed volumetric imaging with relatively low phototoxicity and photobleaching.

#### Confocal microscopy

To overcome some basic limitations of light and fluorescence microscopy, such as tissue penetrance and light scattering, laser scanning confocal microscopy (LSCM) was developed. LSCM provides a means of rejecting the out-of-focus light from the detector such that it does not contribute to the images being collected. The basic principle of confocal microscopy is that the illumination and detection optics are focused on the same diffraction-limited spot, which is moved over the sample to build the complete image on the detector. While the entire field of view is illuminated during confocal imaging, anything outside the focal plane contributes little to the image, lessening the haze observed in standard light microscopy. In LSCM, a laser beam is swept over the sample by means of scanning galvanometer mirrors. The laser is directed onto a pair of scanning mirrors sweeping the beam in x and y directions of a single field of view and then moved incrementally across the entire sample to produce an image of the optical section or slice. To collect a z-stack, the focal point is changed, and the scanning process repeated over the new slice. Upon collection of all optical sections from top to bottom, a 3D image can be reconstructed of the sample. A variation of this method is spinning disk confocal microscopy, which allows for simultaneous scanning of multiple points. Although some spatial resolution is sacrificed compared with LSCM, the improvement in temporal resolution is significant. Spinning disk confocal microscopy allows for high-resolution imaging in thick (typically 1–200μm (8)), 3D tissues and in translucent organisms such as zebrafish (9). A number of common fluorescence-imaging techniques are often combined with confocal microscopy, including FRET (10), fluorescence recovery after photobleaching (11), fluorescence lifetime imaging (12), spectral imaging (13), and optogenetics within live 3D tissue (14). Confocal microscopy can also be useful to study *in vitro* living brain slices for 4D analysis. This was shown by Bredza *et al.* (15) in studies on the brain slices of mice expressing amyloid precursor protein (APP), a model of Alzheimer’s disease (AD). They were able to visualize axons, dendrites, dendritic spines, and dystrophic neurites over many hours. Because of its advantages, ease of use, and relatively low cost, confocal microscopy remains one of the most commonly employed microscopic technologies used to study neurodegeneration in 3D and 4D. However, confocal microscopy is still limited because of its use of high energy illumination to image within brain tissue, which over long periods of time can be phototoxic. As a result, confocal microscopy is often primarily used on fixed tissue.

#### 2P microscopy

2P microscopy was established as an alternative to confocal microscopy. It provides greater tissue penetrance and less background fluorescence than standard epifluorescence and confocal microscopy, enabling more precise imaging of the populations of neurons. It works by illuminating the sample with pulses of long wavelength/low energy photons that achieve a density at the focal plane sufficient to combine to form high energy photons and excite the fluorophores in the optical plane. The use of low energy photons leads to less cellular phototoxicity and so is better tolerated by living systems. Since the low energy photons are incapable of exciting the fluorophore and high energy photons are preferentially generated at the focal plane, there also is less background excitation and emission of the fluorophore from regions of the tissue outside the focal plane. In addition, low energy photons are scattered less than high energy photons by tissue, further reducing unwanted background signal. This results in 3D images of depths of up to 1 mm in brain tissue (16) and means that 2P imaging can be used over longer periods of time with fewer toxic effects. Combined with vascular markers to repeatedly visualize the same field of view, it can provide 4D temporal resolution of neuronal morphology and activity *in vivo* over many months (17).

One of the drawbacks of conventional 2P calcium imaging systems is the limited field of view. To address this limitation, more advanced 2P microscopes were developed having 2 articulated arms to simultaneously image 2 brain areas, either nearby or distal (18, 19). Lecoq *et al.* (19) used 2P microscopes for concurrent Ca^2+^ imaging of neurons in the primary visual cortex and lateromedial visual area in behaving mice. Their studies showed an interaction of neurons between these distal cortical regions in response to visual stimuli, suggesting that sensory responses are propagated through extended cortical networks. The ability to probe brain neuronal activity simultaneously with animal behavior provides an ideal situation to track the onset of neurodegeneration. However, typically, only acute time courses of behavior can be viewed with conventional 2P imaging, which makes it difficult to coordinate with the time course of neurodegeneration.

Another limitation of 2P microscopy is the depth of the tissue in which neuronal activity can be monitored, which prevents imaging of subcortical regions in intact animals. To study subcortical regions, investigators have generally had to remove overlying cortical tissues. For example, Mizrahi *et al.* (20) developed an acute hippocampal window preparation, where the cortex above the hippocampus is removed, to investigate the functional dynamics of dendritic spines in hippocampus *in vivo* (20). However, these methods can produce considerable brain damage which may influence the neuronal activity readouts. To partially overcome these limitations, three-photon microscopy has been developed (21), which uses even longer wavelength and lower energy photon illumination than 2P microscopy to excite a variety of blue and green fluorophores, such as the current generations of protein-based genetically engineered calcium indicators. Three-photon microscopy has been employed to functionally image neurons in regions deep within the cortex and in hippocampus of an intact mouse brain(reference). However, this approach still requires removal of part of the skull of the animal.

#### Miniscope imaging

Another major limitation of the use of 2P microscopy for *in vivo* studies in rodents is that it requires the animal to either be anesthetized or have their head fixed, limiting the range of behaviors that can be monitored. To overcome these limitations, miniaturized head-mounted microscopes (miniscopes) were developed to study neuronal activity *in vivo* in behaving animals (22). When miniscopes are implanted in the brain, they can image neuronal activity from deep brain regions (22). Miniscopes utilize a conventional epifluorescence microscope in which the objective serves to both deliver the excitation light and collect the fluorescence emitted by the specimen to form an image of the brain region of interest.

Activity in neurons expressing fluorescent Ca^2+^ probes can be monitored with miniscopes in the form of short movies that are recorded repetitively over time, allowing for the measurement of the activity of same neuron longitudinally *in vivo*. This was first shown in studies by Ziv *et al.* (23), in which Ca^2+^ imaging was performed in 10 sessions over 45 days in mice trained to run back and forth on a linear track. To longitudinally follow the activity of individual neurons over time, the cells were automatically identified (registered) across all time points in the experiment by modeling the distribution of similarities between neighboring cells across sessions. The authors used this approach to image neuronal activity from the hippocampus and cortex of behaving mice and showed that the same cells within a field of view could be tracked over multiple weeks. Because miniscope imaging can be employed in awake, freely moving animals, it has been used to study the neuronal circuitry involved in a range of behaviors that cannot be studied under head-fixed conditions, such as sleep, narcolepsy, reward seeking and addiction, social behaviors, and feeding.

Miniscope imaging has also been used to study neuronal circuitry in models of epilepsy. Using an established kainic acid model of epilepsy, Berdyyeva *et al.* (24) were able to identify waves of Ca^2+^ activity in the hippocampus that occurred well before the onset of motor convulsions and before any consistent electroencephalography phenotype could be identified. Thus, it is possible that the motor convulsions and electroencephalography signatures that are typically used to study seizures may reflect the latent expression of CNS pathology and result from seizure propagation rather than initiation. Moreover, when valproate, a seizure medication, was given prior to seizure induction, it reduced motor convulsions but failed to alleviate aberrant Ca^2+^ dynamics. This is notable because many candidate epilepsy therapies have failed, possibly because they were developed to ameliorate the symptoms but fail to target the underlying pathology. Integrating Ca^2+^ imaging with concurrent assessments of seizures in animal models may therefore improve the potential of drug candidates to succeed.

The major disadvantage of the miniscope technology is that it is invasive. Miniscope probes are inserted into the brain region of interest, and the extent of damage this inflicts acutely or chronically, and how that alters neuronal activity, is unclear. This can be a problem in studying neurodegenerative disease models because any tissue damage induced by imaging may impact the progression and nature of the disease phenotypes. Furthermore, the field of view of these technologies can be limited. Finally, miniscopes, as well as 2P microscopy, were designed to monitor neuronal activity but are generally not well equipped to quantify neuronal death, a common endpoint in the study of neurodegeneration.

#### Light sheet microscopy

Light sheet fluorescence microscopy (LSFM) is a more recently developed technique to study 3D tissues. In LSFM, the illumination beam is perpendicular to the imaging system, creating a sheet of light through the 2D plane of the sample. This contrasts with typical microscopy techniques, in which the illumination and imaging systems use the same light path and are on the same axis.

LSFM has multiple benefits over confocal microscopy. First, because a single 2D plane can be imaged instead of a point, it can acquire images at speeds at least 100 times faster than those obtained by point-scanning methods such as confocal microscopy. Composite images can be generated within seconds to minutes, as opposed to minutes to hours when using a confocal microscope. Secondly, only one plane is excited, meaning that there is far less out-of-focus light when compared to confocal, which improves signal and creates images with higher contrast. Thirdly, LSFM allows for scanning across a large sample, with the images stitched together digitally to create an image larger than the field of view of the camera. Rotational movement allows researchers to view samples from multiple angles and produce a 3D image. In addition, as only the observed section is illuminated, the sample as a whole receives less light, reducing bleaching, photodamage, and stress and enabling imaging of live cells for much longer time periods.

LSFM has been used to study 3D tissues *in vivo*. Royer *et al.* (25) developed an automated light-sheet microscope that systematically assessed and optimized spatial resolution across living organisms by adapting to the optical properties of the specimen over time. The enhanced 3D imaging is particularly useful to study small organisms such as *Drosophila* and zebrafish. A modified high-speed version of LSFM has also been developed that uses Ca^2+^ indicators to monitor the activity of nearly 80% of the neurons in the zebrafish brain to chart neural circuits with correlated firing patterns (26).

By using a light sheet reflected from a vertically positioned illumination objective to slice horizontally across cultured cell samples, LSFM has also been used to study intracellular pathways and molecules in cells. For example, it was used to directly image the binding of steroid receptor proteins to DNA within living cells. LSFM has also been used to study the location of histone proteins in cells and visualize the assembly of microtubules over the course of mitosis (27).

Despite clear advantages over epifluorescence, confocal, and 2P microscopy in the ability to image broad areas of tissue rapidly and without much harm to the tissue over time, LSFM still suffers from several key limitations. Due to the need for multiple lenses, sample preparation is complex, reducing the number of models that can be imaged. Whereas small model organisms such as zebrafish larvae and small organoid tissues can be easily mounted to image live in a light sheet microscope, other animals and tissue types can be more difficult to mount or immobilize for multiple objectives in a 3D arrangement. Additionally, LSFM can suffer from some of the same constraints of phototoxicity and penetrance of light through opaque tissue, requiring sample fixation and tissue clearing to render the sample transparent and facilitate penetration of light.

#### Robotic microscopy

Another advanced live cell imaging technology specifically designed to study neurodegeneration in 4D is robotic microscopy. Robotic microscopy is an unbiased, sensitive, and quantitative method to longitudinally monitor neurons *in vitro* and *in vivo* with single cell resolution. It has been employed extensively to study neurodegeneration of rodent and human models of neurodegenerative disease in 2D monolayer cultures. It has also been used to investigate cell nonautonomous roles of human glia in neurodegeneration (28). Because of its high throughput and high content capabilities, robotic microscopy has been used for RNAi screening to identify genetic modifiers of various neurodegenerative disease (29) as well as for screening drugs to identify disease-modifying therapeutics (30, 31). Using it, genetic modifiers and mechanisms of neurodegeneration in the models of Parkinson’s disease (PD) (32, 33, 34, 35), amyotrophic lateral sclerosis (ALS) (30, 36, 37), Huntington’s disease (HD) (31, 38, 39, 40, 41) and frontotemporal dementia (29, 42) have been discovered, as well as autophagy inducers that mitigate disease phenotypes.

Robotic microscopy is a fully automated time-lapse confocal imaging technology designed to collect fluorescence images from individual neurons over their lives (38). Using fiducial marks on the bottom of the plate and custom software, it collects information from individual cells by automatically returning to precisely the same microscope field repetitively for days to months, so that fluorescence signals from biosensors introduced into individual cells can be collected at regular intervals and analyzed offline (43). This provides for temporal analysis of neurons because automated analysis programs that can be employed through open-source python packages can segment and identify individual cells in each image, assign them a unique identifying number, track them over the imaging time course, extract fluorescence signals from labels in each cell, and determine cellular characteristics in subsequent images (30, 31, 36, 37, 39, 40, 41, 44). The hardware to execute robotic microscopy is commercially available through vendors that sell automated confocal microscope systems that can be integrated with automated cell culture incubators to achieve automated longitudinal imaging. Customized automated scheduling and microscope control software must be engineered to coordinate imaging throughput between the microscope and automated incubator.

Using fluorescent markers, robotic microscopy can quantify changes over time in neurite area to monitor slow, degenerative processes in neurons. Because each cell is tracked and used as its own control, the impact of cell-to-cell variability on measurements is reduced, resulting in over 100–1000-fold more sensitivity to detect phenotypic differences between two cohorts than more conventional single snapshot approaches (43, 45). With Cox proportional hazards and new Bayesian hierarchical approaches (40, 46, 47), accurate quantitative dynamic predictive models can be constructed, identifying variables directly from complex datasets in parallel, rather than artificially “holding” variables constant (40, 41, 47). This capability, along with a range of unique biosensors, has been used to identify cellular mechanisms that accelerate or slow down degeneration. While mainly used to study neurons in 2D monolayers, robotic microscopy has recently been applied to monitor neuronal dynamics in 4D in rodent and human brain tissue, *in vivo* in zebrafish larvae (48, 49) and in human brain organoids (Fig. 2, *A*–*C*). However, despite its advantages in tracking single cells over time in tissue and within animals during neurodegeneration and its scalability, 3D/4D robotic microscopy is currently still reliant on spinning-disk confocal microscopy and thus suffers from similar drawbacks including low photon efficiency and phototoxicity.

**Figure 2:**
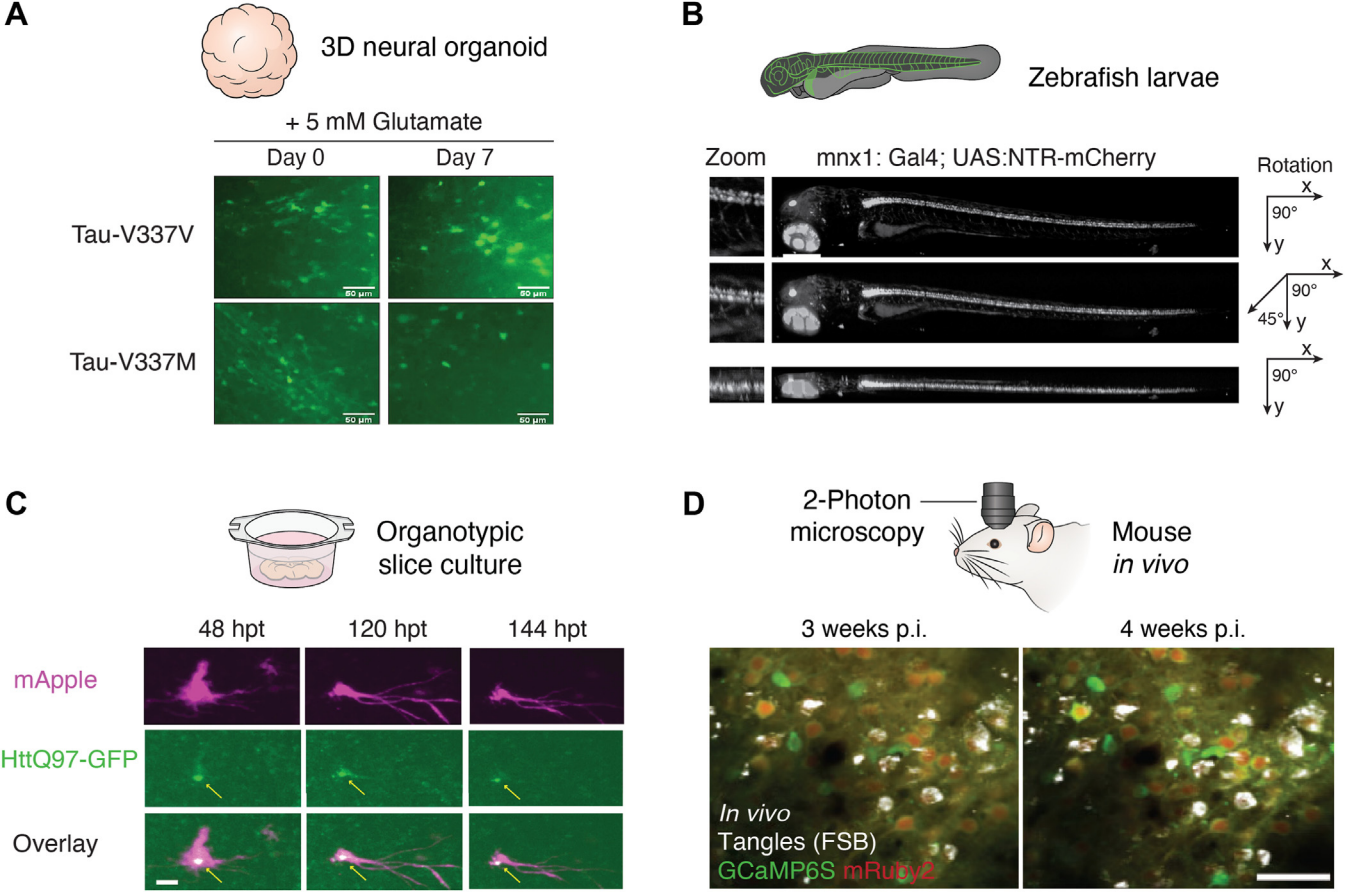
**Biological models used for 3D/4D imaging of neurodegeneration.** A range of biological models can provide 4D imaging insights for neurodegeneration over time. *A*, (*Top*) Cartoon depiction of a 3D neural organoid. (*Bottom*) Z stack maximum reconstruction of Tau-V337M or isogenic-corrected control (Tau-V337V) cerebral organoids transduced with hSyn1:EGFP showing degeneration over time. Scale bar represents 20 μm. Adapted with permission from Bowles *et al.*, (138). *B*, (*Top left*) Cartoon depiction of a whole translucent zebrafish larvae on its side. (*Top right*) Maximum z stack projections imaged with robotic microscopy of a live transgenic zebrafish larvae at 48 h post fertilization showing mCherry-labeled hindbrain neurons before neurodegeneration using metronidazole (MTZ) chemoablation technique. Scale bar represents 400 μm. Adapted with permission from Linsley *et al.* (96). *C*, (*Top*) Cartoon depiction of organotypic slide culture. (*Bottom*) Maximum z stack projections of time lapse imaging of organotypic hippocampal slice culture transfected with mApple and Htt-Ex1-Q97-GFP, a model for Huntington’s disease using a biolistic gene gun. Imaged using robotic microscopy. Adapted with permission from Linsley *et al.* (49). Scale bar represents 300 m. *Arrows* point to the emergence of an inclusion body of Htt-Ex1-Q97-GFP protein. *D*, (*Top*) Cartoon depiction of positioning of *in vivo* 2-photon microscopy. (*Bottom*) 2-photon microscopy of mouse brain, overlay of GCaMP6s and mRuby2, 3 weeks and 4 weeks post injection (p.i.) with misfolded tau fibrils. Adapted with permission from Marinkovic *et al.* (144). Scale bar represents 50 um.

## *In vivo* imaging animal models of neurodegeneration

Animal models—employing a range of different species including *Caenorhabditis elegans*, *Drosophila melanogaster*, zebrafish larvae, and rodents—have been used to study neurodegenerative diseases including HD, PD, and AD. Here, we focus on the vertebrate models of neurodegeneration, but extensive work on *C. elegans* and *D. melanogaster* are reviewed elsewhere (50, 51). To study these diseases, mutant and transgenic animals have been engineered to express genes and their variants believed to be critical for pathogenesis in humans. These models are amenable to analysis using 4D imaging to study the slow cellular and neuronal circuitry changes underlying the pathology of neurodegenerative diseases (Fig. 2).

### Mouse models

**Figure 3:**
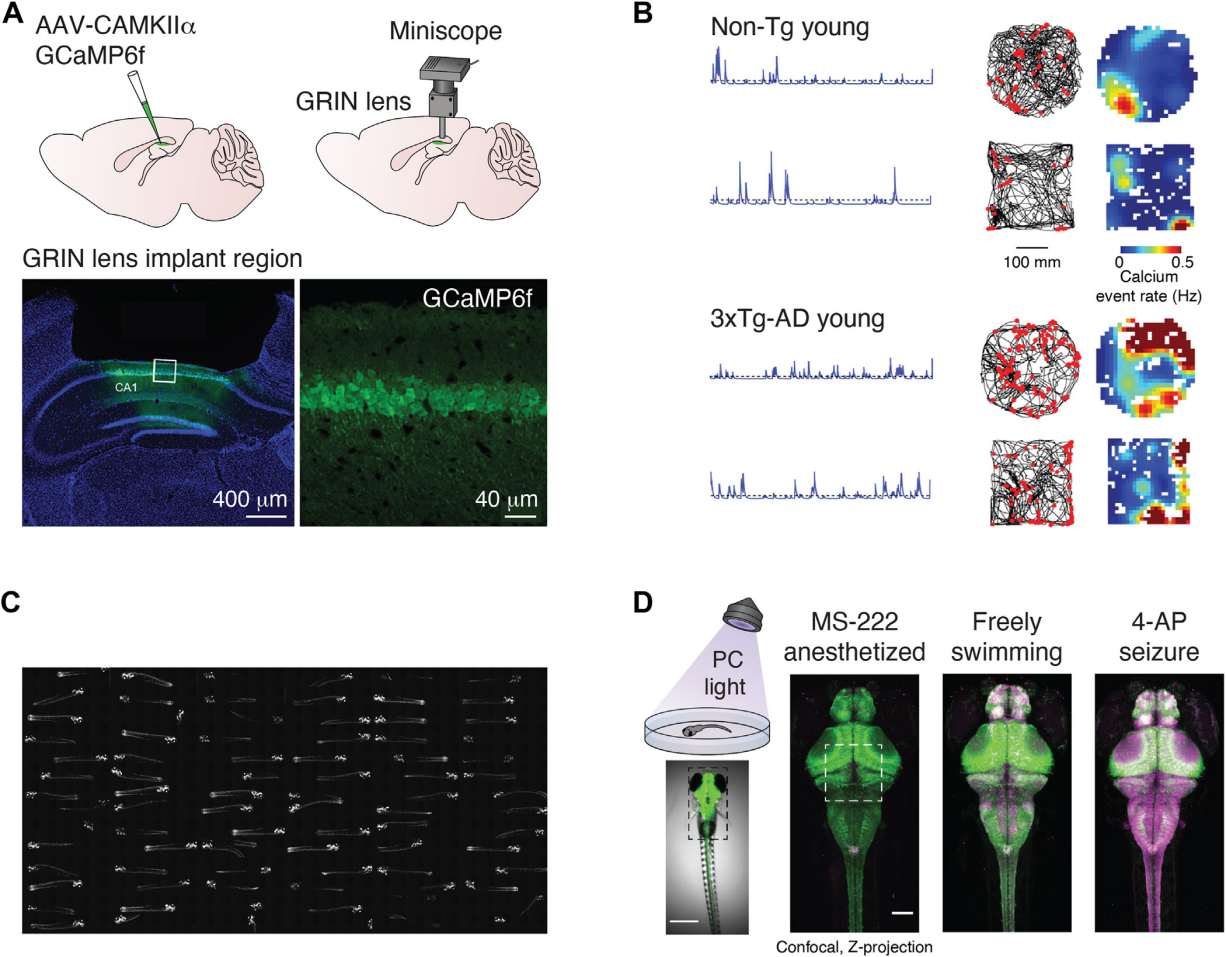
**Analysis of animal behavior paired with 3D/4D imaging.***A*, *in vivo* miniscope calcium imaging setup of transduced mouse hippocampus showing fluorescent GCaMP expression facilitates readout of neuronal activity in awake and active mice. Adapted with permission from Lin *et al.* (145). *B*, paired calcium imaging and behavioral traces from nontransgenic control mouse and 3xTg-AD mice generated from setup in showing neuronal activity and behavioral activity side by side (*A*). *C*, high throughput 4D imaging of live fluorescent zebrafish expressing mCherry throughout the brain. Adapted with permission from Linsley *et al.* (96). *D*, the use of CaMPARI calcium integrator can be paired with high throughput behavioral or imaging to monitor changes and localization of whole brain activity imaged in high throughput in (*C*). Photoconverting (PC) UV flood light triggers the photoconversion of the green fluorescent indicator to *red* and is proportional to the calcium activity levels within the translucent zebrafish brain. Confocal z-projection overlays of the *green* and *red* CaMPARI channels showing differences between anesthetized, freely swimming, and 4-AP seizure-induced animals. Adapted with permission from Fosque *et al.* (99).

Mice are the most commonly used and widely accepted animal models of neurodegeneration (Table 1). However, significant limitations such as the size of their brains and the invasiveness involved in generating *in vivo* data limit the application of 3D/4D studies in mouse models. Nevertheless, paired with analysis of behaviors that mimic common features of human disease, 3D/4D imaging of mouse models can be a powerful tool for modeling the etiology of neurodegenerative disease (Fig. 3, *A* and *B*).

### Huntington’s disease

The neuropathological alterations associated with HD include degeneration and gradual loss of medium spiny neurons (MSNs) in the striatum, a key region of the basal ganglia involved in control of movement. Cortical atrophy and gradual degeneration of cortical-striatal neurons is also prominent in the disease. 4D imaging of mouse HD models has been particularly useful in the investigation of the relationship between the dysfunction of the cortical glutamatergic input to the degeneration of striatal MSNs. Burgold *et al.* (52) used chronic *in vivo* 2P Ca^2+^ imaging to demonstrate that cortical neurons in the 150 CAG repeat R6/2 mouse model of HD (53)—which is characterized by a severe, fast-progressing phenotype—were hyperactive prior to the appearance of motor deficits but then reduced in activity and gradually degenerated. In the less severe 115 CAG repeat R6/1 model of HD, Fernández-García *et al.* (54) used optogenetic techniques to show that stimulation of degenerating secondary motor cortex neurons that project to the dorsolateral striatum reversed motor deficits and normalized spine density within the striatum, supporting the notion that gradual loss of activity of cortical-striatal neurons impacts the degeneration of striatal MSNs in HD. Using 2P microscopy in the R6/2 HD model, Oikonomou *et al.* (55) found altered Ca^2+^ transients and Ca^2+^ buffering capacity in cortical-striatal neurons, which was proposed to impact their function and survival. Hyperactivity followed by degeneration and gradual loss of cortical striatal neurons has been suggested to contribute to dysfunction and gradual degeneration of MSNs in HD, which impairs motricity.

### Parkinson’s disease

Like HD, mouse models of PD have been extensively used to study the impact of impaired basal ganglia neuronal circuitry on disease pathophysiology. Modeling either involves pharmacological perturbations that impact nigrostriatal neuronal health or genetic drivers of disease. Most mouse models feature degeneration and death of nigrostriatal dopaminergic (DA) neurons, a pathological hallmark of PD. 4D imaging studies by Parker *et al.* (56) using miniscope technology investigated how the loss of the DA input produces chronic imbalances in the activity of the direct MSN pathway that projects to the substantia nigra reticulata and indirect MSN pathways projecting to the external globus pallidus (57). They showed that destruction of DA neurons with 6-hydroxydopamine (6-OHDA) altered locomotion and reduced the activity of the direct MSN pathway while increasing the activity of the indirect MSN pathway.

Maltese *et al.* (58) also used 2P imaging to study the striatal direct and indirect MSN pathways and the effect of 6-OHDA lesioning on chronic activity of MSNs. They showed that they could differentially affect the activity of the two MSN populations using selective dopamine D1 or D2 receptor agonists and antagonists. Like the studies from Parker *et al.* (56), Maltese *et al.* (58) also showed that lesioning the nigrostriatal DA neurons causes a major imbalance in the activity of the two MSN pathways, with reduced activity of direct MSNs and increased activity of indirect MSNs. In chronically lesioned animals, treatment with L-DOPA produced an exaggerated increase in the activity of direct MSNs, while the activity of the indirect MSNs was close to basal levels.

4D imaging has also facilitated studies on the impact of genetic factors and acute insults on neurodegeneration in PD. Chen *et al.* (59) showed imbalances in striatal MSNs in PD transgenic models. Mutations in the protein kinase LRRK2, which cause familial forms of PD, disrupt connections between either the direct or indirect MSNs with other neurons in the striatum. Using 2P microscopy in the striatal slices of animals expressing mutant forms of LRRK2, Chen *et al.* (59) showed that the mutations affected how the MSNs respond to inputs from other neurons and reshape synaptic structure and function of these MSNs. This was possible because 2P microscopy can induce repetitive glutamate uncaging to stimulate persistent spine volume increase over time (60), which can be visualized as a change in spine shape under a fluorescence microscope. Together, these studies suggest that the two populations of MSNs respond differently in animals expressing mutations that cause PD and to the loss of DA neuronal input or to excessive DA receptor activity due to L-DOPA treatment. These imbalances may be associated with the motor symptoms in PD patients and those that experience L-DOPA–induced dyskinesias.

In addition to the striatum, cortical dysfunction and atrophy is prominent in PD and occurs before motor symptoms appear. In fact, many of the non-motor symptoms of PD, including cognitive deficit, occur well before the appearance of motor dysfunction and are likely due to altered circuitry in the cortex. Furthermore, alterations in cortical-striatal circuitry could contribute to striatal dysfunction and degeneration. To address these questions, the dynamics of neurocircuitry degeneration must be understood. Guo *et al.* (61) studied the role of DA depletion on synaptic dynamics in the motor cortex using 2P microscopy in mice treated with 6-OHDA or 1-methyl-4-phenyl-1,2,3,6-tetrahydropyridine, which selectively destroy dopaminergic neurons. They showed that DA loss produced dramatic changes in motor cortex dendritic spine dynamics and remodeling, and the reorganization led to unstable neuronal circuits in the motor cortex. They also showed that remodeling was differentially regulated by D1 *versus* D2 DA receptors, with D1 receptor signaling regulating spine elimination, while D2 dopamine receptor signaling was linked to spine formation. DA depletion impaired learning of new motor skills assessed using long term potentiation (LTP) paradigms due to impaired spine dynamics and impairment of learning-induced rewiring stabilization. In control animals, LTP increased dendritic spine densities in the motor cortex, which stabilized over time as assessed by chronic 2P microscopy. In the 1-methyl-4-phenyl-1,2,3,6-tetrahydropyridine-treated mice, LTP induced an increase in dendritic spine density, but these spines were short lived and were eliminated over time. Thus, with 3D/4D imaging, the authors were able to deduce that abnormal spine turnover in the motor cortex is due to impaired neuronal plasticity and may contribute to motor deficits observed in PD.

### Alzheimer’s disease

4D imaging has been particularly useful in helping elucidate the etiological mechanisms in rodent AD models, from pathological mechanisms at the neurocircuitry level down to subcellular biological mechanisms. These technologies have also been employed to investigate the spatiotemporal relationship between synaptic and cognitive impairment and the accumulation of beta-peptide (Aβ) and phosphorylated tau (p-tau), two pathological hallmarks of AD. Bittner *et al.* (62) used 2P microscopy to longitudinally study synaptic changes in triple transgenic AD mice (3xTg-AD), a commonly used AD mouse model which progressively develops both Aβ and tau pathology in the cortex and hippocampus. They found that in young transgenic AD mice, there was a discrete loss of dendritic spines and neurons and an increase in Aβ oligomers selectively in layer III of the cortex. Based on the temporal resolution of dendritic spine loss, they proposed that loss of neurons in this region was due to the toxic actions of soluble Aβ rather than Aβ plaque formation or tau, and may involve a region of neuropathology early in the disease process.

In older 3xTg-AD mice, at a time when amyloid plaques and p-tau are abundant, Bittner *et al.* (62) found that the dendritic spine density declines in proximity to amyloid plaques in a number of cortical and hippocampal regions. However, there is also a reduction in the density of a population of dendritic spines at a distance from amyloid plaques. This plaque-independent spine loss occurred exclusively at dystrophic dendrites that accumulate both soluble Aβ oligomers and p-tau intracellularly. Their 4D imaging studies suggest that there are distinct spatio-temporal patterns of dendritic spine and synaptic loss in the AD mouse model driven by different forms of Aβ and tau. This is of interest because it suggests that therapies designed to selectively reduce Aβ plaques may only partly diminish the progression of neuropathology in AD brain and are particularly of note since 2P microscopy studies by Peters *et al.* (63) showed that tau is a driving force for Aβ plaque formation.

AD neuropathology is also driven by altered brain network activity, due in part to cortical and hippocampal neuronal hyperactivity (64, 65, 66, 67, 68, 69, 70, 71). To investigate the cellular mechanisms of this neuronal hyperactivity, Busche *et al.* (72) used chronic 2P Ca^2+^ imaging *in vivo* to monitor neuronal activity in CA1 neurons of the hippocampus of transgenic mice overexpressing both mutated APP and PS1 in neurons. These mutations are known to induce a rapid increase in soluble brain Aβ levels, followed by the formation of plaques beginning at an early age. They found that in young AD mice, there was a pronounced increase in hippocampal neuronal activity before the appearance of Aβ plaques, and as the animals aged, the hyperactivity continued as the levels of plaques increased, suggesting that the persistent increase in hippocampal neuronal activity may be related to the increase in soluble Aβ oligomers. Consistent with this hypothesis, they found that acutely inhibiting soluble Aβ oligomer formation with the γ-secretase inhibitor LY-411575 reduced soluble Aβ levels and rescued neuronal dysfunction. In addition, direct application of soluble Aβ oligomers to the hippocampus of control WT mice was sufficient to induce neuronal hyperactivity. Thus, these 3D/4D imaging studies suggest that soluble Aβ oligomers may be critical drivers of early hippocampal hyperactivity and network dysfunction.

Similar findings were reported by Sosulina *et al.* (73) using 2P Ca^2+^-imaging *in vivo* in the hippocampus of a rat model of AD overexpressing APP, the precursor of Aβ. They proposed that increased intrinsic excitability of CA1 neurons occurs at an early stage of Aβ-deposition and disease progression. Other studies have shown that common pathophysiological consequences of higher neuronal excitability are structural alterations such as reduced dendritic arborization, spine loss, synapse loss, and reduced cell size (62, 74, 75, 76, 77).

4D imaging at the subcellular level within tissue to track aggregation of tau proteins has also been achieved. With 2P microscopy, fluorescence recovery after photobleaching, and the photoactivatable biosensor Dendra2, Wegmann *et al.* (78) showed the formation of droplet-like tau protein in mouse cortex undergoing liquid–liquid phase separation *in vivo*. The ability to track subcellular and intracellular phenomena within a live animal is an exemplary demonstration of the promise of 3D/4D imaging and reveals the extraordinary power of tracking subcellular phenomena within tissue.

A number of 4D imaging studies have helped elucidate the role of non-neuronal cells such as microglia and astrocytes in the pathogenesis of AD. It has long been noted that microglia and astrocytes cluster within or around Aβ plaques in patients and mouse models of AD (79, 80). 4D studies in the mouse models of AD have shown that the time course of plaque formation and microglia movement can be followed with 2P microscopy, helping to parse the progression of the disease (81, 82). Meyer-Luehmann *et al.* (82) demonstrated that microglia are recruited to plaques within days of the plaque’s appearance and can clear the plaques if fully activated. In contrast, other studies (7, 81) showed that microglia can remain at the interface with plaques for weeks to months, suggesting a role for microglia in plaque maintenance.

Additional 4D studies have been used to define potential therapeutic targets within microglia towards AD. Garcia-Alloza *et al.* (83) found that microglia did not appear to effectively remove plaques on their own within the mouse brain without the administration of anti-Aβ immunotherapy which directed microglia towards senile plaques and led to a reduction of plaque size. Fuhrmann *et al.* (84) showed using 2P imaging in mouse model brains that microglia migrate and increase in number around neurons that are subsequently lost, suggesting microglia play a key role in neuronal death. Additionally, they demonstrated that knockdown of the G protein–coupled chemokine receptor CX3CR1, which is expressed in microglia, disrupts neuron-microglia communication, and prevents this neuronal loss. More recent studies by Fuger *et al.* (85) showed that many microglia are long-lived and persist over the course of most of an animal’s life in normal conditions. Yet in AD model mice, microglia loss and proliferation are increased at least three-fold compared to a WT control, suggesting that the increase in microglia surrounding plaques results from abnormal proliferation of microglia in plaque-free areas.

The role of astrocytes in the development of AD remains somewhat more mysterious. Several studies have noted altered morphology and calcium dynamics in astrocytes within AD mouse models. Galea *et al.* (86) demonstrated with 2P 4D imaging that astrocytes undergo phenotypic changes such as GFAP upregulation and development of a “reactive” appearance in response to the presence of amyloid plaques but do not migrate towards plaques in AD model mouse brains, instead maintaining their highly organized domains. In contrast, Kuchibhotla *et al.* (87) used 4D imaging to record calcium homeostasis disruptions within the brain-wide astrocytic network in AD mouse models, demonstrating that plaques do indeed have an astrocyte-based network effect on the brain. Additional studies are needed to determine whether these astrocytic phenotypes are pathological drivers or represent homeostatic mechanisms that compensate for disease progression. Overall, these studies demonstrate that the ability to track the interactions between neurons and other cell types within a live brain in response to disease using 4D imaging will continue to play a valuable role in defining the pathogenesis and the roles of potential therapeutics in AD.

### Amyotrophic lateral sclerosis

The neuromuscular junction, which plays a key role in the pathogenesis of ALS, represents a complex connection of nerve and muscle fibers that is difficult to model with cell culture in two dimensions (88). As a result, 4D imaging of mouse neuromuscular junctions has often been used to track the progression of disease in ALS models such as the well-characterized SOD1-G93A mouse. Using confocal and 2P microscopy, Dibaj *et al.* (89) investigated inflammatory activity over time in the SOD1-G93A mouse. They found differences of responses of fluorescently labeled microglia in the CNS and macrophages in the peripheral nervous system during disease onset, providing new insight into how each contributes to the pathology of the disease. Using *in vivo* confocal imaging of SOD1-G37R mice, Martineau *et al.* (90) showed that dynamic remodeling of the neuromuscular junction takes place early in disease progression and represents a complex interplay of denervation and new innervation rather than a sudden global denervation of the motor neuron. To look at subcellular dynamics of ALS, Bilsland *et al.* (91) used time lapse confocal microscopy to image axonal transport defects in the motor neurons of SOD1-G93A mice, finding that disrupted retrograde axonal transport may represent one of the earliest pathologies in the progression of the disease. In each of these cases, the use of 4D imaging within a fully intact neuromuscular junction in a mouse model of ALS garnered a key insight into the pathogenesis of the disease.

### Zebrafish models

Zebrafish have been employed to model a number of neurodegenerative diseases. Approximately 84% of genes known to be associated with human neurodegenerative disease have a zebrafish counterpart (92), including *SMA1*, *HTT*, *Tau*, *TDP43*, and *APP*. Zebrafish have a number of characteristics that make them versatile animal models for 3D/4D imaging, including their rapid development *ex utero* and large reproductive capacity, which facilitates experiments at larger scale and with greater statistical power than can be achieved with rodents. Zebrafish are genetically tractable (93), show a diverse repertoire of stereotypical behaviors, and drug treatment is easy and convenient in the aqueous environment (94). But perhaps the greatest advantage of zebrafish as a model organism is their translucent skin, which facilitates noninvasive optical imaging *in vivo* and optogenetic stimulation (95). Zebrafish larvae are uniquely suited for longitudinal imaging *in vivo* because, until ∼10 days postfertilization (dpf), they are fed off of their yolk sacs, allowing them to be pharmacologically immobilized from 2 to 10 dpf without dying. Finally, the entire zebrafish larvae nervous system can be imaged longitudinally, making them a particularly appealing model for the studies of neural circuitry and neurodegeneration.

Longitudinal *in vivo* imaging of the activity of neurons in the zebrafish larvae is possible using robotic microscopy and Ca^2+^ biosensors such as GCaMP7 (96). Precise and sensitive monitoring of neurodegeneration and death can be achieved with genetically engineered calcium indicators using robotic microscopy, which provides an automated, high content, 4D longitudinal single-cell tracking microscopy platform for longitudinal imaging of live zebrafish larvae *in toto* over multiple days. In this approach, automated confocal microscopy can be used to repeatedly image each fish in three dimensions at specified intervals, generating 4D images of each fish in an array.

Another advantage of zebrafish is their fecundity and scalability for behavioral assays. They have often been employed in behavioral high-throughput drug screens to discover therapeutics to treat CNS diseases. As altered behaviors are clinical hallmarks of all neurodegenerative diseases, monitoring behavior in neurodegenerative disease models can be particularly effective and elucidate sources of neurodegeneration within the CNS when used to complement 3D/4D *in vivo* imaging (97, 98, 99) (Fig. 3, *C* and *D*). A range of behaviors can be monitored in zebrafish in relation to changes in CNS activity, including locomotor activity (100, 101) and their response to visual, tactile, or acoustic stimuli (102, 103). They also move in response to alternating light and dark conditions as a measure of anxiety-like behavior, which is a common symptom in neurodegenerative diseases such as AD (104, 105, 106). Zebrafish can be subjected to cognitive tests including spatial discrimination in the T/Y-maze as a measure of learning and memory (107) and exploratory activities (108, 109). They have also been employed to classify complex psychoactive compounds, and the results were shown to be predictive of compound activity in mice (110).

### Parkinson’s disease

3D/4D imaging of zebrafish has been useful in elucidating mechanisms of degeneration in PD. For example, O’Donnell *et al.* (111) investigated the causative role of aSYN on PD. They expressed human aSYN fused to GFP using a ribosomal skipping P2A peptide in zebrafish Rohon-Beard neurons, which are peripheral neurons in the developing spinal cord that project sensory axons to the skin. Both the cell bodies and the elaborate peripheral arbors of these cells were monitored *in vivo* by confocal microscopy and time-lapse analysis, permitting visualization of axonal transport and degeneration. Cell death was quantified by counting cells expressing aSYN-2A-GFP in the embryos every 20 to 60 min for up to 12 h, and the images were compiled into movies. These studies showed that human aSYN could induce neurodegeneration in the zebrafish peripheral sensory neurons. Taking advantage of the intact architecture of the *in vivo* brain, the authors also showed that the axonal compartment of neurons is more vulnerable than the cell body to aSYN toxicity. Defects in axonal mitochondria morphology and transport, especially in the anterograde direction, were detected, as was the eventual accumulation of the organelles in axonal varicosities.

Others have applied machine learning to analyze zebrafish behavior to gain insight into PD. For example, Hughes *et al.* (112) examined behavior in zebrafish bearing a loss-of-function mutation in PARK7 that causes a rare form of early onset PD. They found that training evolutionary machine learning algorithms with the continuous data stream of animal behavior mitigated bias and allowed for the computation of more wide-ranging features to discriminate PD models from control zebrafish. The ability to discern and quantify a parkinsonian behavior can facilitate large scale behavior-based screens of zebrafish PARK7 mutants to identify therapeutics and therapeutic targets that could mitigate those behaviors while also contextualizing the effect within the whole zebrafish brain. The ability to combine 3D imaging with behavioral output in zebrafish neurodegeneration models at scale holds great promise for the dissection of the disrupted neurocircuitry during neurodegeneration and for the identification of novel therapeutic targets (Fig. 3, *C* and *D*).

### Alzheimer’s disease

Transgenic AD zebrafish have also been developed to overexpress tau to induce neuropathology. Lopez *et al.* (113) developed zebrafish models of tauopathy by generating a transgenic line overexpressing human A152T-tau (2N4R) mutation. They showed that the levels of tau phosphorylation were increased in animals with mutant tau. Using live animal confocal microscopy, they showed abnormal branching and numbers of motor neurons in the zebrafish expressing the mutant tau compared to the WT tau. They also showed the mutant tau induced behavioral deficits in escape responses. Furthermore, using the photoswitchable fluorescent protein Dendra fused to tau, the authors monitored tau clearance kinetics in individual spinal cord motor neurons *in vivo* and showed that A152T-tau was cleared more slowly than WT tau due to impaired proteasome activity. This finding provides important translational insight, as it suggests that therapeutically targeting autophagy could improve clearance of A152T-tau and reduce its toxicity.

## Mouse organotypic culture system

Organotypic brain cultures are commonly used to study rodent brain neurons *in vitro* in 3D. In contrast to acute cultures that are typically used and discarded over a period of hours, organotypic cultures are maintained in culture medium over periods of days to months. Organotypic cultures provide distinct advantages over dissociated neurons because they maintain the cytoarchitecture of brain regions, preserving neuronal circuitry and interaction with non-neuronal cell types such as astrocytes and microglia. Neurons from organotypic cultures are also believed to have a more mature state than neurons in 2D culture, especially when derived from more developed or aged brains (114). Organotypic cultures can also be maintained and imaged for much longer periods of time (up to 6 months) than dissociated cells or acute brain slices (115). The longitudinal nature of the system is an advantage for investigating neurodegeneration, which is generally a slow, gradual process. The cultures are also amenable to genetic manipulations to either express disease-causing proteins in neurons of interest or express fluorescent probes for imaging those cells (116).

Organotypic cultures of the hippocampus as well as other brain regions maintain electrical properties comparable to those observed in acute slices (117). Furthermore, synapses mature in culture towards a phenotype comparable to adult brain (118). Developmental changes in spine density and shape and increased connectivity recapitulate the *in vivo* phenotype observed in acute slices from age-matched time points (118). Various other regions of the brain, including cortex, cerebellum, and thalamus, have also been successfully cultured. In addition, co-cultures of various brain regions including the nigrostriatal circuit (119), the cortex, and hippocampus (114, 116), as well as entire sagittal and coronal organotypic cultures (120) have been reported, each of which can be used as a model for neurodegenerative disease. Recent work has shown that organotypic cultures can be prepared from aged mice, providing unique insight into aged neurons (121), and can be seeded with misfolded pathogenic proteins such as tau to study the propagation of misfolded protein throughout the tissue (122).

One advantage of organotypic slice culture is the ability to combine electrophysiological methods with imaging. The patch-clamp method has been used to study individual neurons, but this approach is time-limited, usually to just 48 h (118, 123). Extracellular recordings and long-term electrical stimulations have been performed by means of multi-electrode arrays (MEAs) (124), which generally measure the activity of populations of neurons. Gong *et al.* (125) developed a high-definition MEA to measure single cell action potentials with high spatiotemporal resolution. They were able to record from individual neurons in the slices of hippocampal organotypic cultures daily for a month. They also developed methods for global network-wide electrical-activity maps of the slice cultures based on spike amplitudes detected across all electrodes on the array.

Long-term optical imaging of organotypic slice cultures has been used to investigate synaptic morphology and connectivity using a variety of techniques, including widefield microscopy, in which fluorescent indicators of cell morphology or activity are expressed in the neurons using adenovirus vectors or in transgenic mice from which the cultures are derived (126). For example, Bodea *et al.* (127) employed time lapse imaging methods to map neurons in ventral midbrain, including DA neurons, in organotypic cultures.

Another advantage of organotypic slice culture is its accessibility and scale for long-term imaging studies. For example, Croft *et al.* (128) showed that turnover and inclusion formation of tau fused to the photoconvertible tag Dendra2 can be monitored longitudinally in 4D. When organotypic slice cultures transduced with a Dendra2-tau biosensor were seeded with misfolded tau fibrils (K18), photoconverted Dendra2-tau persisted and could be tracked over several weeks in culture.

Another imaging technology used to monitor neurons in organotypic cultures is robotic microscopy. This longitudinal technology was recently used to monitor in a fully automated manner the daily morphological changes within individual neurons in 4D in organotypic cultures from rodent brain and human primary brain tissue for at least 3 weeks (49). It was able to track changes in cell number, velocity, morphology, position, and neuronal health on a protracted scale. 4D-robotic microscopy is high throughput and also high content, capable of analyzing 70 brain slices at a time and monitoring the slow processes of neurodegeneration in 3D tissues. As an example, the mutant protein that causes HD, mHtt, was expressed in neurons in organotypic cultures of rodent brain along with a morphology marker, mApple. 4D robotic microscopy was able to detect the slow accumulation of aggregates of mHtt in neurons as well as the subsequent gradual loss of neurons (49).

Organotypic cultures of rodent hippocampus have been particularly useful in studies on the uptake and spread of pathogenic proteins in brain in the models of AD and PD. For example, Ortiz-Sanz *et al.* (129) employed 2P microscopy to study the synaptic effects and changes in spine morphology induced by exogenously applied Aβ oligomers on hippocampal organotypic cultures. In parallel *in vivo* studies, time-lapse imaging showed that Aβ oligomers significantly decreased the stability of spines, and the acute effects of Aβ oligomers on spines occurred *via* mechanisms involving CaMKII and integrin β1 activities. Similarly, Shrivastava *et al.* (130) identified specific structures of aSYN fibrils taken up into neurons in organotypic hippocampal slice cultures from WT mice and showed that the uptake and aggregation of aSYN fibrils alters neuronal network activity monitored by MEA recordings. These studies suggested the uptake of monomeric aSYN, and its aggregation in neurons affects neuronal homeostasis and produces changes in function and activity.

## Human organoid models

In recent years, patient i-neurons have been used to model human neurodegenerative diseases. These models have advantages over non-human models because they can be generated directly from human patients and thus fully recapitulate the human genetic background of the disease. This is important, because for most human neurodegenerative diseases, multiple genes and their variants contribute to neuropathology. Furthermore, most disease models based on non-human cells are dependent on the expression of mutant genes that cause disease in only a small population of patients. In contrast, human i-neurons can be generated from patients with sporadic forms of disease, providing models of neurodegeneration that represent the majority of patients rather than the select few with familial forms of the disease.

In addition to 2D systems, i-neurons can be grown into 3D organoids to recapitulate some of the circuitry and heterotypic interactions found *in vivo*. A number of technological advances have been made to improve the environmental complexity of neuronal organoids, including co-emergence of astrocytes and microglia which support neuronal function and survival (131, 132), as well as methods to enhance synaptogenesis and spontaneous synaptic activity. Pașca *et al.* (133) developed novel 3D culture systems using human i-neurons that better represent the laminated neuronal structure in human brain. They could be maintained for up to 9 months *in vitro* and reliably expressed genes resembling those found in the developing human cortex during the late to mid-fetal period. These cultures expressed markers of multiple distinct classes of cells including those for forebrain neurons and glia. When sectioned and analyzed morphologically, the organoids showed similar cytoarchitectures to those found in developing human cortex. By immunocytochemistry, it was also possible to distinguish superficial and deep cortical layers, which developed proportionally.

The use of organoids for studying neurodegeneration is in its infancy but is already showing great promise. One advantage is that neuronal organoids appear to mature better than 2D iPSC cultures and thus may better model late-onset neurodegenerative disease. For example, single cell and single nuclei transcriptomics analyses and electrophysiological analyses of organoids show cell phenotypes that more closely match those found in an adult brain (134, 135, 136).

The power of 4D imaging of the human organoid culture system was nicely shown in studies by Sakaguchi *et al.* (137), who investigated network activity in human cerebral cortical organoids using time-lapse confocal imaging. Sakaguchi *et al.* monitored Ca^2+^ imaging over short intervals and showed the neural networks expressed synchronized burst activity, and some neurons showed spontaneous activity. Neuron survival can also be tracked within organoids derived from patients with neurodegenerative disease. With longitudinal confocal imaging, Bowles *et al.* (138) tracked survival of fluorescently labeled neurons in organoids derived from patients with dementia-related mutations and gene-corrected controls. They found neurons containing disease-associated tau mutations survived less well than controls. Furthermore, pharmacological rescue was shown to increase survival, demonstrating the power of organoids to model disease and the effects of candidate therapies (138).

A challenge to the use of neuronal organoids is that because of their thickness, it can be difficult to image them at depth without first clearing the tissue. Furthermore, as they grow larger, inner regions of organoids do not receive sufficient oxygenation to allow for proper neuronal development, function, and survival. To address this issue, Giandomenico *et al.* (139) employed an air-liquid interface (ALI) technique to increase oxygen supply and improve neuronal survival and axon outgrowth. Similar to organotypic slice culture, ALI facilitated and simplified long-term live imaging, allowing visualization of GFP-labeled neurons and greatly improving survival and morphology, with extensive axon outgrowths reminiscent of nerve tracts. The use of ALI organoids combined with 4D imaging holds great promise for the investigations of neurodegenerative disease etiology.

## Challenges and future directions

The application of novel imaging technologies to study neurodegeneration often lags a step behind other biomedical research because of the unique challenges to imaging neurodegeneration. For one thing, new animal and tissue-based models of neurodegeneration that replicate the etiologies of these complex diseases are continuously in development (140), and additional work is required to make each model imaging-compatible. In particular, more work needs to be done to correlate and adapt 4D microscopy technologies to newly developed large animal and nonhuman primate models of neurodegeneration which often model many features of human pathophysiology but typically rely on 4D positron emission tomography imaging techniques that lack cellular resolution (1). Additional challenges are often encountered when identifying the ideal time course, time resolution, and fields of view to analyze a phenotype of interest, especially in neurodegenerative disease models where tissues typically require maturity or aging for degeneration to occur. Nevertheless, it will be exciting to see if other cutting-edge live microscopy technologies such as adaptive optics (25), quantitative phase contrast (141), 3D super resolution (142), and ever complex iterations of light sheet microscopy (143), currently gaining increasing use in developmental biology, can be adapted to meet the challenges of studying neurodegenerative diseases.

Another area for improvement in the 3D/4D imaging of neurodegeneration is in development of fluorescent biosensors and analysis algorithms to help interpret the complex, high content signals coming from cells (Table 2). In principle, many if not all genetically encoded biosensors that have been used in 2D cell culture could be implemented in 3D/4D imaging to help detect subcellular phenomena within tissue. Indeed, more and more biosensors are being incorporated into such assays and can appreciably impact the insight gathered in the studies of neurodegenerative disease. In live imaging studies of neurodegeneration, a particularly acute challenge is precisely determining whether a particular neuron is alive, dead, or dying, since dyes and stains used to distinguish live from dead neurons in culture may be unable to detect the onset of cell death. Furthermore, in live imaging experiments, the loss of fluorescence of transfected neurons, indicating the rupture of the plasma membrane, has been shown to clearly mark neuronal death, but fluorescent debris often persists for days after initial morphological signs of death and decay occur, limiting the ability to identify the precise time of death or introducing human error in the scoring of neuronal death by morphology. For these reasons, a biosensor to detect the “point of no return,” at which a neuron’s fate is unambiguously sealed, has been needed to determine the cellular mechanisms that lead up to neuronal death.

**Table 2:** Commonly used biosensors for 3D/4D studies of neurodegeneration

Biosensor	Common use	References
Morphology (GFP, mApple)	Fluorescent labeling of neurons, neuronal morphology	(17, 49, 111, 138, 139)
GCaMP	Calcium imaging	(19, 23, 24, 26, 49, 52, 55, 72, 73, 137)
CaMPARI	Large scale calcium dynamics integration	(98)
Dendra fusions (Dendra-Tau, Dendra-synuclein)	Protein turnover dynamics	(113, 128)
GEDI	Detection of cell death/degeneration	(96)
Cry2-TDP43	Optogenetic-mediated TDP43 oligomerization, aggregation	(95)

The genetically encoded fluorescent cell-death indicator (GEDI) was recently developed to monitor with ∼100% accuracy the very earliest time point of neurodegeneration, when a neuron commits to die but is still alive (96). GEDI specifically detects intracellular Ca^2+^ levels that are only achieved when cells are irreversibly committed to die and has been used to enable an automated imaging platform for single cell detection of neuronal death *in vivo* in zebrafish larvae. With a quantitative ratiometric signal, GEDI facilitates high throughput analysis of cell death in time-lapse imaging data, providing the resolution and scale to identify early factors, such as autophagy-lysosomal dysfunction, leading to cell death in the studies of neurodegeneration. Since neurodegeneration can be asynchronous, the greater sensitivity of GEDI allows researchers to identify neurodegeneration *in vivo* in discrete neuronal populations, providing previously unobtainable delimitation and clarity to the time course of cell death.

Additional biosensors capable of informing on cellular and subcellular phenotypes that are particularly designed for challenges present in 3D/4D imaging data such as GEDI are much needed. There is currently a dearth of biosensors developed to track neuroimmune responses, transmission of misfolded proteins, and effects on brain neurocircuitry, despite the rising interest in these processes as mechanisms of neurodegeneration. Biosensors capable of informing these studies, integrating with new animal and tissue models of neurodegeneration, and that function with the latest microscopy technologies, will help lead the way forward in understanding the huge complexity of these diseases.

## Conflict of interest

S. F. is the inventor of Robotic Microscopy Systems, US Patent 7,139,415 and Automated Robotic Microscopy Systems, US Patent Application 14/737,325, both assigned to the J. David Gladstone Institutes. A provisional US and EPO patent for the GEDI biosensor (inventors J. W. L. and S. F.) assigned to the J. David Gladstone Institutes has been placed GL2016 to 815, May 2019. S. F. and J. W. L. are co-founders of Operant Biopharma. The other author declares that they have no conflicts of interest with the contents of this article.

## References

[1] Chen B., Marquez-Nostra B., Belitzky E., Toyonaga T., Tong J., Huang Y. (2022). PET imaging in animal models of Alzheimer's disease. Front. Neurosci..

[2] Vasilkovska T., Adhikari M.H., Van Audekerke J., Salajeghe S., Pustina D., Cachope R. (2023). Resting-state fMRI reveals longitudinal alterations in brain network connectivity in the zQ175DN mouse model of Huntington's disease. Neurobiol. Dis..

[3] Hafkemeijer A., Möller C., Dopper E.G., Jiskoot L.C., Schouten T.M., van Swieten J.C. (2015). Resting state functional connectivity differences between behavioral variant frontotemporal dementia and Alzheimer's disease. Front. Hum. Neurosci..

[4] Jalilianhasanpour R., Beheshtian E., Sherbaf G., Sahraian S., Sair H.I. (2019). Functional connectivity in neurodegenerative disorders: Alzheimer's disease and frontotemporal dementia. Top Magn. Reson. Imaging.

[5] Moguilner S., García A.M., Perl Y.S., Tagliazucchi E., Piguet O., Kumfor F. (2021). Dynamic brain fluctuations outperform connectivity measures and mirror pathophysiological profiles across dementia subtypes: a multicenter study. Neuroimage.

[6] Fan L.Z., Kheifets S., Böhm U.L., Wu H., Piatkevich K.D., Xie M.E. (2020). All-optical electrophysiology reveals the role of lateral inhibition in sensory processing in cortical layer 1. Cell.

[7] Hefendehl J.K., Wegenast-Braun B.M., Liebig C., Eicke D., Milford D., Calhoun M.E. (2011). Long-term *in vivo* imaging of β-amyloid plaque appearance and growth in a mouse model of cerebral β-amyloidosis. J. Neurosci..

[8] Wang S., Larina I.V., Narayan R.J. (2017). Monitoring and Evaluation of Biomaterials and Their Performance In Vivo.

[9] Muto A., Kawakami K. (2013). Prey capture in zebrafish larvae serves as a model to study cognitive functions. Front. Neural Circuits.

[10] Kardash E., Bandemer J., Raz E. (2011). Imaging protein activity in live embryos using fluorescence resonance energy transfer biosensors. Nat. Protoc..

[11] Linsley J.W., Hsu I.U., Groom L., Yarotskyy V., Lavorato M., Horstick E.J. (2017). Congenital myopathy results from misregulation of a muscle Ca2+ channel by mutant Stac3. Proc. Natl. Acad. Sci. U. S. A..

[12] Andrews N., Ramel M.C., Kumar S., Alexandrov Y., Kelly D.J., Warren S.C. (2016). Visualising apoptosis in live zebrafish using fluorescence lifetime imaging with optical projection tomography to map FRET biosensor activity in space and time. J. Biophotonics.

[13] Høgset H., Horgan C.C., Armstrong J.P.K., Bergholt M.S., Torraca V., Chen Q. (2020). In vivo biomolecular imaging of zebrafish embryos using confocal Raman spectroscopy. Nat. Commun..

[14] Zhu P., Narita Y., Bundschuh S.T., Fajardo O., Schärer Y.P., Chattopadhyaya B. (2009). Optogenetic dissection of neuronal circuits in zebrafish using viral gene transfer and the Tet system. Front. Neural Circuits.

[15] Brendza R.P., Simmons K., Bales K.R., Paul S.M., Goldberg M.P., Holtzman D.M. (2003). Use of YFP to study amyloid-beta associated neurite alterations in live brain slices. Neurobiol. Aging.

[16] Benninger R.K.P., Piston D.W. (2013). Two-photon excitation microscopy for the study of living cells and tissues. Curr. Protoc. Cell Biol..

[17] Gu L., Kleiber S., Schmid L., Nebeling F., Chamoun M., Steffen J. (2014). Long-term *in vivo* imaging of dendritic spines in the hippocampus reveals structural plasticity. J. Neurosci..

[18] Stirman J.N., Smith I.T., Kudenov M.W., Smith S.L. (2016). Wide field-of-view, multi-region, two-photon imaging of neuronal activity in the mammalian brain. Nat. Biotechnol..

[19] Lecoq J., Savall J., Vučinić D., Grewe B.F., Kim H., Li J.Z. (2014). Visualizing mammalian brain area interactions by dual-axis two-photon calcium imaging. Nat. Neurosci..

[20] Mizrahi A., Crowley J.C., Shtoyerman E., Katz L.C. (2004). High-resolution *in vivo* imaging of hippocampal dendrites and spines. J. Neurosci..

[21] Akbari N., Tatarsky R.L., Kolkman K.E., Fetcho J.R., Bass A.H., Xu C. (2022). Whole-brain optical access in a small adult vertebrate with two- and three-photon microscopy. iScience.

[22] Stamatakis A.M., Resendez S.L., Chen K.S., Favero M., Liang-Guallpa J., Nassi J.J. (2021). Miniature microscopes for manipulating and recording *in vivo* brain activity. Microscopy (Oxf).

[23] Ziv Y., Burns L.D., Cocker E.D., Hamel E.O., Ghosh K.K., Kitch L.J. (2013). Long-term dynamics of CA1 hippocampal place codes. Nat. Neurosci..

[24] Berdyyeva T.K., Frady E.P., Nassi J.J., Aluisio L., Cherkas Y., Otte S. (2016). Direct imaging of hippocampal epileptiform calcium motifs following kainic acid administration in freely behaving mice. Front. Neurosci..

[25] Royer L.A., Lemon W.C., Chhetri R.K., Wan Y., Coleman M., Myers E.W. (2016). Adaptive light-sheet microscopy for long-term, high-resolution imaging in living organisms. Nat. Biotechnol..

[26] Ahrens M.B., Orger M.B., Robson D.N., Li J.M., Keller P.J. (2013). Whole-brain functional imaging at cellular resolution using light-sheet microscopy. Nat. Methods.

[27] Chen B.C., Legant W.R., Wang K., Shao L., Milkie D.E., Davidson M.W. (2014). Lattice light-sheet microscopy: imaging molecules to embryos at high spatiotemporal resolution. Science.

[28] Serio A., Bilican B., Barmada S.J., Ando D.M., Zhao C., Siller R. (2013). Astrocyte pathology and the absence of non-cell autonomy in an induced pluripotent stem cell model of TDP-43 proteinopathy. Proc. Natl. Acad. Sci. U. S. A..

[29] Elia L.P., Mason A.R., Alijagic A., Finkbeiner S. (2019). Genetic regulation of neuronal progranulin reveals a critical role for the autophagy-lysosome pathway. J. Neurosci..

[30] Barmada S.J., Serio A., Arjun A., Bilican B., Daub A., Ando D.M. (2014). Autophagy induction enhances TDP43 turnover and survival in neuronal ALS models. Nat. Chem. Biol..

[31] Tsvetkov A.S., Miller J., Arrasate M., Wong J.S., Pleiss M.A., Finkbeiner S. (2010). A small-molecule scaffold induces autophagy in primary neurons and protects against toxicity in a Huntington disease model. Proc. Natl. Acad. Sci. U. S. A..

[32] Nakamura K., Nemani V.M., Azarbal F., Skibinski G., Levy J.M., Egami K. (2011). Direct membrane association drives mitochondrial fission by the Parkinson disease-associated protein α-synuclein. J. Biol. Chem..

[33] Skibinski G., Hwang V.D., Ando D.M., Daub A., Lee A.K., Ravisankar A. (2016). Nrf2 mitigates LRRK2- and α-synuclein-induced neurodegeneration by modulating proteostasis. Proc. Natl. Acad. Sci. U. S. A..

[34] Skibinski G., Nakamura K., Cookson M.R., Finkbeiner S. (2014). Mutant LRRK2 toxicity in neurons depends on LRRK2 levels and synuclein but not kinase activity or inclusion bodies. J. Neurosci..

[35] Li H., Doric Z., Berthet A., Jorgens D.M., Nguyen M.K., Hsieh I. (2021). Longitudinal tracking of neuronal mitochondria delineates PINK1/Parkin-dependent mechanisms of mitochondrial recycling and degradation. Sci. Adv..

[36] Barmada S., Finkbeiner S. (2010). Bringing SOD1 into the fold. Nat. Neurosci..

[37] Barmada S.J., Ju S., Arjun A., Batarse A., Archbold H.C., Peisach D. (2015). Amelioration of toxicity in neuronal models of amyotrophic lateral sclerosis by hUPF1. Proc. Natl. Acad. Sci. U. S. A..

[38] Arrasate M., Mitra S., Schweitzer E.S., Segal M.R., Finkbeiner S. (2004). Inclusion body formation reduces levels of mutant huntingtin and the risk of neuronal death. Nature.

[39] Miller J., Arrasate M., Shaby B.A., Mitra S., Masliah E., Finkbeiner S. (2010). Quantitative relationships between huntingtin levels, polyglutamine length, inclusion body formation, and neuronal death provide novel insight into Huntington‘s disease molecular pathogenesis. J. Neurosci..

[40] Miller J., Arrasate M., Brooks E., Libeu C.P., Legleiter J., Hatters D. (2011). Identifying polyglutamine protein species *in situ* that best predict neurodegeneration. Nat. Chem. Biol..

[41] Tsvetkov A.S., Arrasate M., Barmada S., Ando D.M., Sharma P., Shaby B.A. (2013). Proteostasis of polyglutamine varies among neurons and predicts neurodegeneration. Nat. Chem. Biol..

[42] Elia L.P., Reisine T., Alijagic A., Finkbeiner S. (2020). Approaches to develop therapeutics to treat frontotemporal dementia. Neuropharmacology.

[43] Arrasate M., Finkbeiner S. (2005). Automated microscope system for determining factors that predict neuronal fate. Proc. Natl. Acad. Sci. U. S. A..

[44] Chang Y.H., Linsley J., Lamstein J., Kalra J., Epstein I., Barch M. (2020). Single cell tracking based on Voronoi partition *via* stable matching. bioRxiv.

[45] Finkbeiner S., Frumkin M., Kassner P.D. (2015). Cell-based screening: extracting meaning from complex data. Neuron.

[46] Aron R., Tsvetkov A., Finkbeiner S. (2013). NUB1 snubs huntingtin toxicity. Nat. Neurosci..

[47] Shaby B.A., Skibinsk G., Ando M., LaDow E.S., Finkbeiner S. (2016). A three-groups model for high-throughput survival screens. Biometrics.

[48] Linsley J.W., Reisine T., Finkbeiner S. (2019). Cell death assays for neurodegenerative disease drug discovery. Expert Opin. Drug Discov..

[49] Linsley J.W., Tripathi A., Epstein I., Schmunk G., Mount E., Campioni M. (2019). Automated four-dimensional long term imaging enables single cell tracking within organotypic brain slices to study neurodevelopment and degeneration. Commun. Biol..

[50] Wolozin B., Gabel C., Ferree A., Guillily M., Ebata A. (2011). Watching worms whither: modeling neurodegeneration in C. elegans. Prog. Mol. Biol. Transl. Sci..

[51] Bolus H., Crocker K., Boekhoff-Falk G., Chtarbanova S. (2020). Modeling neurodegenerative disorders in Drosophila melanogaster. Int. J. Mol. Sci..

[52] Burgold J., Schulz-Trieglaff E.K., Voelkl K., Gutiérrez-Ángel S., Bader J.M., Hosp F. (2019). Cortical circuit alterations precede motor impairments in Huntington's disease mice. Sci. Rep..

[53] Mangiarini L., Sathasivam K., Seller M., Cozens B., Harper A., Hetherington C. (1996). Exon 1 of the HD gene with an expanded CAG repeat is sufficient to cause a progressive neurological phenotype in transgenic mice. Cell.

[54] Fernández-García S., Conde-Berriozabal S., García-García E., Gort-Paniello C., Bernal-Casas D., García-Díaz Barriga G. (2020). M2 cortex-dorsolateral striatum stimulation reverses motor symptoms and synaptic deficits in Huntington's disease. Elife.

[55] Oikonomou K.D., Donzis E.J., Bui M.T.N., Cepeda C., Levine M.S. (2021). Calcium dysregulation and compensation in cortical pyramidal neurons of the R6/2 mouse model of Huntington's disease. J. Neurophysiol..

[56] Parker J.G., Marshall J.D., Ahanonu B., Wu Y.W., Kim T.H., Grewe B.F. (2018). Diametric neural ensemble dynamics in parkinsonian and dyskinetic states. Nature.

[57] Gerfen C.R., Engber T.M., Mahan L.C., Susel Z., Chase T.N., Monsma F.J. (1990). D1 and D2 dopamine receptor-regulated gene expression of striatonigral and striatopallidal neurons. Science.

[58] Maltese M., March J.R., Bashaw A.G., Tritsch N.X. (2021). Dopamine differentially modulates the size of projection neuron ensembles in the intact and dopamine-depleted striatum. Elife.

[59] Chen C., Soto G., Dumrongprechachan V., Bannon N., Kang S., Kozorovitskiy Y. (2020). Pathway-specific dysregulation of striatal excitatory synapses by LRRK2 mutations. Elife.

[60] Matsuzaki M., Honkura N., Ellis-Davies G.C., Kasai H. (2004). Structural basis of long-term potentiation in single dendritic spines. Nature.

[61] Guo L., Xiong H., Kim J.I., Wu Y.W., Lalchandani R.R., Cui Y. (2015). Dynamic rewiring of neural circuits in the motor cortex in mouse models of Parkinson's disease. Nat. Neurosci..

[62] Bittner T., Fuhrmann M., Burgold S., Ochs S.M., Hoffmann N., Mitteregger G. (2010). Multiple events lead to dendritic spine loss in triple transgenic Alzheimer's disease mice. PLoS One.

[63] Peters F., Salihoglu H., Pratsch K., Herzog E., Pigoni M., Sgobio C. (2019). Tau deletion reduces plaque-associated BACE1 accumulation and decelerates plaque formation in a mouse model of Alzheimer's disease. EMBO J..

[64] Bakker A., Krauss G.L., Albert M.S., Speck C.L., Jones L.R., Stark C.E. (2012). Reduction of hippocampal hyperactivity improves cognition in amnestic mild cognitive impairment. Neuron.

[65] Dickerson B.C., Salat D.H., Greve D.N., Chua E.F., Rand-Giovannetti E., Rentz D.M. (2005). Increased hippocampal activation in mild cognitive impairment compared to normal aging and AD. Neurology.

[66] Sperling R.A., Laviolette P.S., O'Keefe K., O'Brien J., Rentz D.M., Pihlajamaki M. (2009). Amyloid deposition is associated with impaired default network function in older persons without dementia. Neuron.

[67] Filippini N., MacIntosh B.J., Hough M.G., Goodwin G.M., Frisoni G.B., Smith S.M. (2009). Distinct patterns of brain activity in young carriers of the APOE-epsilon4 allele. Proc. Natl. Acad. Sci. U. S. A..

[68] Bookheimer S.Y., Strojwas M.H., Cohen M.S., Saunders A.M., Pericak-Vance M.A., Mazziotta J.C. (2000). Patterns of brain activation in people at risk for Alzheimer’s disease. N. Engl. J. Med..

[69] Quiroz Y.T., Budson A.E., Celone K., Ruiz A., Newmark R., Castrillon G. (2010). Hippocampal hyperactivation in presymptomatic familial Alzheimer's disease. Ann. Neurol..

[70] Sepulveda-Falla D., Glatzel M., Lopera F. (2012). Phenotypic profile of early-onset familial Alzheimer's disease caused by presenilin-1 E280A mutation. J. Alzheimers Dis..

[71] Palop J.J., Mucke L. (2016). Network abnormalities and interneuron dysfunction in Alzheimer disease. Nat. Rev. Neurosci..

[72] Busche M.A., Chen X., Henning H.A., Reichwald J., Staufenbiel M., Sakmann B. (2012). Critical role of soluble amyloid-β for early hippocampal hyperactivity in a mouse model of Alzheimer's disease. Proc. Natl. Acad. Sci. U. S. A..

[73] Sosulina L., Mittag M., Geis H.R., Hoffmann K., Klyubin I., Qi Y. (2021). Hippocampal hyperactivity in a rat model of Alzheimer's disease. J. Neurochem..

[74] Bittner T., Burgold S., Dorostkar M.M., Fuhrmann M., Wegenast-Braun B.M., Schmidt B. (2012). Amyloid plaque formation precedes dendritic spine loss. Acta Neuropathol..

[75] Grutzendler J., Gan W.B. (2007). Long-term two-photon transcranial imaging of synaptic structures in the living brain. CSH Protoc..

[76] Moolman D.L., Vitolo O.V., Vonsattel J.P., Shelanski M.L. (2004). Dendrite and dendritic spine alterations in Alzheimer models. J. Neurocytol..

[77] Tsai J., Grutzendler J., Duff K., Gan W.B. (2004). Fibrillar amyloid deposition leads to local synaptic abnormalities and breakage of neuronal branches. Nat. Neurosci..

[78] Wegmann S., Eftekharzadeh B., Tepper K., Zoltowska K.M., Bennett R.E., Dujardin S. (2018). Tau protein liquid-liquid phase separation can initiate tau aggregation. EMBO J..

[79] Perlmutter L.S., Barron E., Chui H.C. (1990). Morphologic association between microglia and senile plaque amyloid in Alzheimer's disease. Neurosci. Lett..

[80] Itagaki S., McGeer P.L., Akiyama H., Zhu S., Selkoe D. (1989). Relationship of microglia and astrocytes to amyloid deposits of Alzheimer disease. J. Neuroimmunol.

[81] Bolmont T., Haiss F., Eicke D., Radde R., Mathis C.A., Klunk W.E. (2008). Dynamics of the microglial/amyloid interaction indicate a role in plaque maintenance. J. Neurosci..

[82] Meyer-Luehmann M., Spires-Jones T.L., Prada C., Garcia-Alloza M., de Calignon A., Rozkalne A. (2008). Rapid appearance and local toxicity of amyloid-beta plaques in a mouse model of Alzheimer's disease. Nature.

[83] Garcia-Alloza M., Borrelli L.A., Thyssen D.H., Hickman S.E., El Khoury J., Bacskai B.J. (2013). Four-dimensional microglia response to anti-Aβ treatment in APP/PS1xCX3CR1/GFP mice. Intravital.

[84] Fuhrmann M., Bittner T., Jung C.K., Burgold S., Page R.M., Mitteregger G. (2010). Microglial Cx3cr1 knockout prevents neuron loss in a mouse model of Alzheimer's disease. Nat. Neurosci..

[85] Füger P., Hefendehl J.K., Veeraraghavalu K., Wendeln A.C., Schlosser C., Obermüller U. (2017). Microglia turnover with aging and in an Alzheimer's model *via* long-term *in vivo* single-cell imaging. Nat. Neurosci..

[86] Galea E., Morrison W., Hudry E., Arbel-Ornath M., Bacskai B.J., Gómez-Isla T. (2015). Topological analyses in APP/PS1 mice reveal that astrocytes do not migrate to amyloid-β plaques. Proc. Natl. Acad. Sci. U. S. A..

[87] Kuchibhotla K.V., Lattarulo C.R., Hyman B.T., Bacskai B.J. (2009). Synchronous hyperactivity and intercellular calcium waves in astrocytes in Alzheimer mice. Science.

[88] Verma S., Khurana S., Vats A., Sahu B., Ganguly N.K., Chakraborti P. (2022). Neuromuscular junction dysfunction in amyotrophic lateral sclerosis. Mol. Neurobiol..

[89] Dibaj P., Steffens H., Zschüntzsch J., Nadrigny F., Schomburg E.D., Kirchhoff F. (2011). In vivo imaging reveals distinct inflammatory activity of CNS microglia *versus* PNS macrophages in a mouse model for ALS. PLoS One.

[90] Martineau É., Di Polo A., Vande Velde C., Robitaille R. (2018). Dynamic neuromuscular remodeling precedes motor-unit loss in a mouse model of ALS. Elife.

[91] Bilsland L.G., Sahai E., Kelly G., Golding M., Greensmith L., Schiavo G. (2010). Deficits in axonal transport precede ALS symptoms *in vivo*. Proc. Natl. Acad. Sci. U. S. A..

[92] Howe K., Clark M.D., Torroja C.F., Torrance J., Berthelot C., Muffato M. (2013). The zebrafish reference genome sequence and its relationship to the human genome. Nature.

[93] Newman M., Nornes S., Martins R.N., Lardelli M.T. (2012). Robust homeostasis of Presenilin1 protein levels by transcript regulation. Neurosci. Lett..

[94] Kokel D., Bryan J., Laggner C., White R., Cheung C.Y., Mateus R. (2010). Rapid behavior-based identification of neuroactive small molecules in the zebrafish. Nat. Chem. Biol..

[95] Asakawa K., Handa H., Kawakami K. (2020). Optogenetic modulation of TDP-43 oligomerization accelerates ALS-related pathologies in the spinal motor neurons. Nat. Commun..

[96] Linsley J.W., Shah K., Castello N., Chan M., Haddad D., Doric Z. (2021). Genetically encoded cell-death indicators (GEDI) to detect an early irreversible commitment to neurodegeneration. Nat. Commun..

[97] Vaz M., Silvestre S. (2020). Alzheimer's disease: recent treatment strategies. Eur. J. Pharmacol..

[98] Biechele-Speziale D., Camarillo M., Martin N.R., Biechele-Speziale J., Lein P.J., Plavicki J.S. (2023). Assessing CaMPARI as new approach methodology for evaluating neurotoxicity. Neurotoxicology.

[99] Fosque B.F., Sun Y., Dana H., Yang C.T., Ohyama T., Tadross M.R. (2015). Neural circuits. Labeling of active neural circuits *in vivo* with designed calcium integrators. Science.

[100] Wang Y., Balaji V., Kaniyappan S., Krüger L., Irsen S., Tepper K. (2017). The release and trans-synaptic transmission of Tau *via* exosomes. Mol. Neurodegener..

[101] Naini S.M.A., Yanicostas C., Hassan-Abdi R., Blondeel S., Bennis M., Weiss R.J. (2020). Correction to: surfen and oxalyl surfen decrease tau hyperphosphorylation and mitigate neuron deficits *in vivo* in a zebrafish model of tauopathy. Transl. Neurodegener..

[102] Cosacak M.I., Bhattarai P., Bocova L., Dzewas T., Mashkaryan V., Papadimitriou C. (2017). Human TAU(P301L) overexpression results in TAU hyperphosphorylation without neurofibrillary tangles in adult zebrafish brain. Sci. Rep..

[103] Pinho B.R., Reis S.D., Hartley R.C., Murphy M.P., Oliveira J.M.A. (2019). Mitochondrial superoxide generation induces a parkinsonian phenotype in zebrafish and huntingtin aggregation in human cells. Free Radic. Biol. Med..

[104] Gomoll B.P., Kumar A. (2015). Managing anxiety associated with neurodegenerative disorders. F1000Prime Rep..

[105] Pan H., Zhang J., Wang Y., Cui K., Cao Y., Wang L. (2019). Linarin improves the dyskinesia recovery in Alzheimer's disease zebrafish by inhibiting the acetylcholinesterase activity. Life Sci..

[106] Barbereau C., Yehya A., Silhol M., Cubedo N., Verdier J.M., Maurice T. (2020). Neuroprotective brain-derived neurotrophic factor signaling in the TAU-P301L tauopathy zebrafish model. Pharmacol. Res..

[107] Pu Y.Z., Liang L., Fu A.L., Liu Y., Sun L., Li Q. (2017). Generation of Alzheimer's disease transgenic zebrafish expressing human APP mutation under control of zebrafish appb promotor. Curr. Alzheimer Res..

[108] Nunes M.E., Müller T.E., Braga M.M., Fontana B.D., Quadros V.A., Marins A. (2017). Chronic treatment with paraquat induces brain injury, changes in antioxidant defenses system, and modulates behavioral functions in zebrafish. Mol. Neurobiol..

[109] Zanandrea R., Abreu M.S., Piato A., Barcellos L.J.G., Giacomini A. (2018). Lithium prevents scopolamine-induced memory impairment in zebrafish. Neurosci. Lett..

[110] Bruni G., Rennekamp A.J., Velenich A., McCarroll M., Gendelev L., Fertsch E. (2016). Zebrafish behavioral profiling identifies multitarget antipsychotic-like compounds. Nat. Chem. Biol..

[111] O'Donnell K.C., Lulla A., Stahl M.C., Wheat N.D., Bronstein J.M., Sagasti A. (2014). Axon degeneration and PGC-1α-mediated protection in a zebrafish model of α-synuclein toxicity. Dis. Model Mech..

[112] Hughes G.L., Lones M.A., Bedder M., Currie P.D., Smith S.L., Pownall M.E. (2020). Machine learning discriminates a movement disorder in a zebrafish model of Parkinson's disease. Dis. Model Mech..

[113] Lopez A., Lee S.E., Wojta K., Ramos E.M., Klein E., Chen J. (2017). A152T tau allele causes neurodegeneration that can be ameliorated in a zebrafish model by autophagy induction. Brain.

[114] Croft C.L., Futch H.S., Moore B.D., Golde T.E. (2019). Organotypic brain slice cultures to model neurodegenerative proteinopathies. Mol. Neurodegener..

[115] Baraban S.C., Southwell D.G., Estrada R.C., Jones D.L., Sebe J.Y., Alfaro-Cervello C. (2009). Reduction of seizures by transplantation of cortical GABAergic interneuron precursors into Kv1.1 mutant mice. Proc. Natl. Acad. Sci. U. S. A..

[116] Duff K., Noble W., Gaynor K., Matsuoka Y. (2002). Organotypic slice cultures from transgenic mice as disease model systems. J. Mol. Neurosci..

[117] Finley M., Fairman D., Liu D., Li P., Wood A., Cho S. (2004). Functional validation of adult hippocampal organotypic cultures as an *in vitro* model of brain injury. Brain Res..

[118] De Simoni A., Griesinger C.B., Edwards F.A. (2003). Development of rat CA1 neurones in acute *versus* organotypic slices: role of experience in synaptic morphology and activity. J. Physiol..

[119] Daviaud N., Garbayo E., Lautram N., Franconi F., Lemaire L., Perez-Pinzon M. (2014). Modeling nigrostriatal degeneration in organotypic cultures, a new *ex vivo* model of Parkinson's disease. Neuroscience.

[120] Staal J.A., Alexander S.R., Liu Y., Dickson T.D., Vickers J.C. (2011). Characterization of cortical neuronal and glial alterations during culture of organotypic whole brain slices from neonatal and mature mice. PLoS One.

[121] Schommer J., Schrag M., Nackenoff A., Marwarha G., Ghribi O. (2017). Method for organotypic tissue culture in the aged animal. MethodsX.

[122] McCarthy J.M., Virdee J., Brown J., Ursu D., Ahmed Z., Cavallini A. (2021). Development of P301S tau seeded organotypic hippocampal slice cultures to study potential therapeutics. Sci. Rep..

[123] Dong H.W., Buonomano D.V. (2005). A technique for repeated recordings in cortical organotypic slices. J. Neurosci. Methods.

[124] Killian N.J., Vernekar V.N., Potter S.M., Vukasinovic J. (2016). A device for long-term perfusion, imaging, and electrical interfacing of brain tissue *in vitro*. Front. Neurosci..

[125] Gong W., Senčar J., Bakkum D.J., Jäckel D., Obien M.E., Radivojevic M. (2016). Multiple single-unit long-term tracking on organotypic hippocampal slices using high-density microelectrode arrays. Front. Neurosci..

[126] Schwarz N., Uysal B., Welzer M., Bahr J.C., Layer N., Löffler H. (2019). Long-term adult human brain slice cultures as a model system to study human CNS circuitry and disease. Elife.

[127] Bodea G.O., Blaess S. (2012). Organotypic slice cultures of embryonic ventral midbrain: a system to study dopaminergic neuronal development *in vitro*. J. Vis. Exp..

[128] Croft C.L., Goodwin M.S., Ryu D.H., Lessard C.B., Tejeda G., Marrero M. (2021). Photodynamic studies reveal rapid formation and appreciable turnover of tau inclusions. Acta Neuropathol..

[129] Ortiz-Sanz C., Gaminde-Blasco A., Valero J., Bakota L., Brandt R., Zugaza J.L. (2020). Early effects of Aβ oligomers on dendritic Spine dynamics and arborization in hippocampal neurons. Front. Synaptic Neurosci..

[130] Shrivastava A.N., Bousset L., Renner M., Redeker V., Savistchenko J., Triller A. (2020). Differential membrane binding and seeding of distinct α-synuclein fibrillar polymorphs. Biophys. J..

[131] Ormel P.R., Vieira de Sá R., van Bodegraven E.J., Karst H., Harschnitz O., Sneeboer M.A.M. (2018). Microglia innately develop within cerebral organoids. Nat. Commun..

[132] Hasselmann J., Blurton-Jones M. (2020). Human iPSC-derived microglia: a growing toolset to study the brain's innate immune cells. Glia.

[133] Pașca S.P. (2018). The rise of three-dimensional human brain cultures. Nature.

[134] Gordon A., Yoon S.J., Tran S.S., Makinson C.D., Park J.Y., Andersen J. (2021). Long-term maturation of human cortical organoids matches key early postnatal transitions. Nat. Neurosci..

[135] Camp J.G., Badsha F., Florio M., Kanton S., Gerber T., Wilsch-Bräuninger M. (2015). Human cerebral organoids recapitulate gene expression programs of fetal neocortex development. Proc. Natl. Acad. Sci. U. S. A..

[136] Yakoub A.M. (2019). Cerebral organoids exhibit mature neurons and astrocytes and recapitulate electrophysiological activity of the human brain. Neural Regen. Res..

[137] Sakaguchi H., Ozaki Y., Ashida T., Matsubara T., Oishi N., Kihara S. (2019). Self-organized synchronous calcium transients in a cultured human neural network derived from cerebral organoids. Stem Cell Rep..

[138] Bowles K.R., Silva M.C., Whitney K., Bertucci T., Berlind J.E., Lai J.D. (2021). ELAVL4, splicing, and glutamatergic dysfunction precede neuron loss in MAPT mutation cerebral organoids. Cell.

[139] Giandomenico S.L., Mierau S.B., Gibbons G.M., Wenger L.M.D., Masullo L., Sit T. (2019). Cerebral organoids at the air-liquid interface generate diverse nerve tracts with functional output. Nat. Neurosci..

[140] Tello J.A., Williams H.E., Eppler R.M., Steinhilb M.L., Khanna M. (2022). Animal models of neurodegenerative disease: recent advances in fly highlight innovative approaches to drug discovery. Front. Mol. Neurosci..

[141] Nguyen T.H., Kandel M.E., Rubessa M., Wheeler M.B., Popescu G. (2017). Gradient light interference microscopy for 3D imaging of unlabeled specimens. Nat. Commun..

[142] Tønnesen J., Inavalli V., Nägerl U.V. (2018). Super-resolution imaging of the extracellular space in living brain tissue. Cell.

[143] Zhao F., Zhu L., Fang C., Yu T., Zhu D., Fei P. (2020). Deep-learning super-resolution light-sheet add-on microscopy (Deep-SLAM) for easy isotropic volumetric imaging of large biological specimens. Biomed. Opt. Express.

[144] Marinković P., Blumenstock S., Goltstein P.M., Korzhova V., Peters F., Knebl A. (2019). *In vivo* imaging reveals reduced activity of neuronal circuits in a mouse tauopathy model. Brain.

[145] Lin X., Chen L., Baglietto-Vargas D., Kamalipour P., Ye Q., LaFerla F.M. (2022). Spatial coding defects of hippocampal neural ensemble calcium activities in the triple-transgenic Alzheimer's disease mouse model. Neurobiol. Dis..

